# Evaluating Age and Growth Relationship to Ciguatoxicity in Five Coral Reef Fish Species from French Polynesia

**DOI:** 10.3390/md20040251

**Published:** 2022-04-01

**Authors:** Hélène Taiana Darius, Christelle Paillon, Gérard Mou-Tham, André Ung, Philippe Cruchet, Taina Revel, Jérôme Viallon, Laurent Vigliola, Dominique Ponton, Mireille Chinain

**Affiliations:** 1 Institut Louis Malardé (ILM), Laboratory of Marine Biotoxins, UMR 241-EIO (IFREMER, ILM, IRD, Université de Polynésie Française), P.O. Box 30, Papeete 98713, Tahiti, French Polynesia; aung@ilm.pf (A.U.); pcruchet@ilm.pf (P.C.); trevel@ilm.pf (T.R.); jviallon@ilm.pf (J.V.); mchinain@ilm.pf (M.C.); 2 ENTROPIE, IRD-Université de la Réunion-CNRS-Université de la Nouvelle-Calédonie-IFREMER, Labex Corail, 98848 Nouméa, New Caledonia, France; christelle.paillon@gmail.com (C.P.); moutham.g@gmail.com (G.M.-T.); laurent.vigliola@ird.fr (L.V.); 3 ENTROPIE, IRD-Université de la Réunion-CNRS-Université de la Nouvelle-Calédonie-IFREMER, c/o Institut Halieutique et des Sciences Marines (IH.SM), Université de Toliara, Rue Dr. Rabesandratana, P.O. Box 141, Toliara 601, Madagascar; dominique.ponton@ird.fr

**Keywords:** ciguatoxins, coral reef fish, neuroblastoma cell-based assay, otolithometry, toxicity determinants

## Abstract

Ciguatera poisoning (CP) results from the consumption of coral reef fish or marine invertebrates contaminated with potent marine polyether compounds, namely ciguatoxins. In French Polynesia, 220 fish specimens belonging to parrotfish (*Chlorurus microrhinos*, *Scarus forsteni*, and *Scarus ghobban*), surgeonfish (*Naso lituratus*), and groupers (*Epinephelus polyphekadion*) were collected from two sites with contrasted risk of CP, i.e., Kaukura Atoll versus Mangareva Island. Fish age and growth were assessed from otoliths’ yearly increments and their ciguatoxic status (negative, suspect, or positive) was evaluated by neuroblastoma cell-based assay. Using permutational multivariate analyses of variance, no significant differences in size and weight were found between negative and suspect specimens while positive specimens showed significantly greater size and weight particularly for *E. polyphekadion* and *S. ghobban*. However, eating small or low-weight specimens remains risky due to the high variability in size and weight of positive fish. Overall, no relationship could be evidenced between fish ciguatoxicity and age and growth characteristics. In conclusion, size, weight, age, and growth are not reliable determinants of fish ciguatoxicity which appears to be rather species and/or site-specific, although larger fish pose an increased risk of poisoning. Such findings have important implications in current CP risk management programs.

## 1. Introduction

Ciguatera poisoning (CP) is the most common seafood-borne poisoning worldwide [1]. Though originally endemic to tropical and intertropical regions, CP cases are now more commonly reported in semi-temperate areas in countries such as the Macaronesian Islands [2,3,4,5] or in the coast of continental countries such as India [6,7]. With an estimated 50,000 to 500,000 yearly cases worldwide, CP highest incidence rates (IRs) are consistently reported in endemic areas, i.e., the Caribbean Sea, and the Pacific and Indian Oceans [1,8,9]. In the Pacific, French Polynesia is among the top 10 Pacific Island Countries and Territories (PICTs) displaying the highest IRs since 1998 [8,9], with an annual mean IR of 159 ± 51 cases/100,000 inhabitants between 2007 and the present day [10]. Fortunately, this disease which is characterized by gastrointestinal, neurological, and cardiovascular disorders, is rarely fatal [1,11].

This seafood poisoning results from the bioaccumulation and biotransformation in marine food webs of neurotoxins known as ciguatoxins (CTXs) originally produced by benthic dinoflagellates belonging to the genera *Gambierdiscus* and *Fukuyoa* [12]. Natural or anthropogenic disturbances in coral reef ecosystems are believed to be triggering factors of ciguatera [13]. Different CTX analogs can be found in *Gambierdiscus*, fish, marine invertebrates, and sharks (Table S1) [12,14,15,16,17]. This pattern of CTX diversity is also combined with increasing potency for some CTX analogs toward higher trophic levels, from herbivorous to carnivorous fish [13,18]. Based on the food chain theory, larger (and thus older) specimens in a given fish species are also expected to be more toxic than smaller ones due to higher CTX accumulation all along their life history [13,19,20]. In the light of heavy reliance of PICTs on a seafood-based diet, their populations are particularly vulnerable to CP [13] and being able to identify determinants of fish ciguatoxicity could truly benefit CP risk assessment programs in areas where this disease is endemic.

The detection of ciguatoxic fish remains very challenging as CTXs are odorless, colorless, and tasteless [13]. Their presence in trace amounts (nanograms) in contaminated matrices also requires access to efficient, highly sensitive laboratory tests with detection limits approaching the very low guidance levels recommended by the US Food and Drug Administration (US FDA) [21]. A concentration of 0.01 µg equivalents of CTX1B kg^−1^ of fish is expected not to exert effects in sensitive individuals [22]. Since no field test kit is available yet for immediate use by local communities and fishermen, local populations in PICTs often turn to a variety of folk tests [23,24] or strategies (avoidance consumption of too big portions, large carnivorous fish, specific parts of the fish, or certain species) to limit the risk of exposure to ciguatera toxins [13]. In some countries, current risk management regulatory practices include bans on the sale of high-risk fish species [25,26,27,28], those from known toxic locations [28], or specimens over a certain size [29,30,31,32,33,34] or weight [5,35,36,37]. In the Canary Islands, data derived from large-scale surveys performed on random wild fish specimens were tentatively used to establish a predictive score of ciguatoxicity in fish [5,37]. Factors such as fish size or weight, trophic levels, lipid content, fishing site, as well as the season of the year were arguably determined as risk factors associated with the probability of catching fish containing CTXs (Table S2). However, due to the contradictory results obtained, no general recommendation currently exists. One possible reason for this lack of correlation is that although the size and weight of fish are expected to reflect its age and growth, decoupling between these life history traits is sometimes observed. For instance, fast-growing fish can reach a large size at a young age, while slow-growing fish will reach the same size at a later age. For species with variable inter-individual growth, large age differences can therefore exist between fish of the same size. Due to the well-known asymptotic pattern of fish growth, these differences may increase for large individuals that are approaching their maximum size, and yet are continuing to age. In this regard, it is speculated that age and growth could be better proxies than size or weight as determinants of fish ciguatoxicity.

The main goal of the present research was therefore to examine the relationship between the ciguatoxicity and age and growth, along with size and weight, in five coral reef fish species known to be at high risk of CP in French Polynesia [10]: namely (i) in herbivores, the steephead parrotfish *Chlorurus microrhinos*, Forsten’s parrotfish *Scarus forsteni*, bluebarred parrotfish *Scarus ghobban* (Scaridae) and orangespine unicornfish *Naso lituratus* (Acanthuridae); and (ii) in carnivores, the marbled grouper *Epinephelus polyphekadion* (Serranidae). Fish specimens were collected from two sites with contrasted risk of CP, namely Mangareva Island (Gambier Archipelago) and Kaukura Atoll (Tuamotu Archipelago). To this end, the ciguatoxic status of 220 herbivorous and carnivorous fish specimens was assessed using the neuroblastoma cell-based assay (CBA-N2a) designed for the detection of voltage-gated sodium channel activators such as CTXs [38]. In parallel, age and growth were estimated through the analysis of seasonal marks on otoliths. Growth back-calculation models (BCM), non-linear mixed-effects models (NLME), and permutational multivariate analysis of variance (PERMANOVA) were then performed, searching for the probability to find CTX-like toxicity in fish according to their size, weight, age, and growth.

## 2. Results and Discussion

### 2.1. Fish Demographic Data

**Figure 1:**
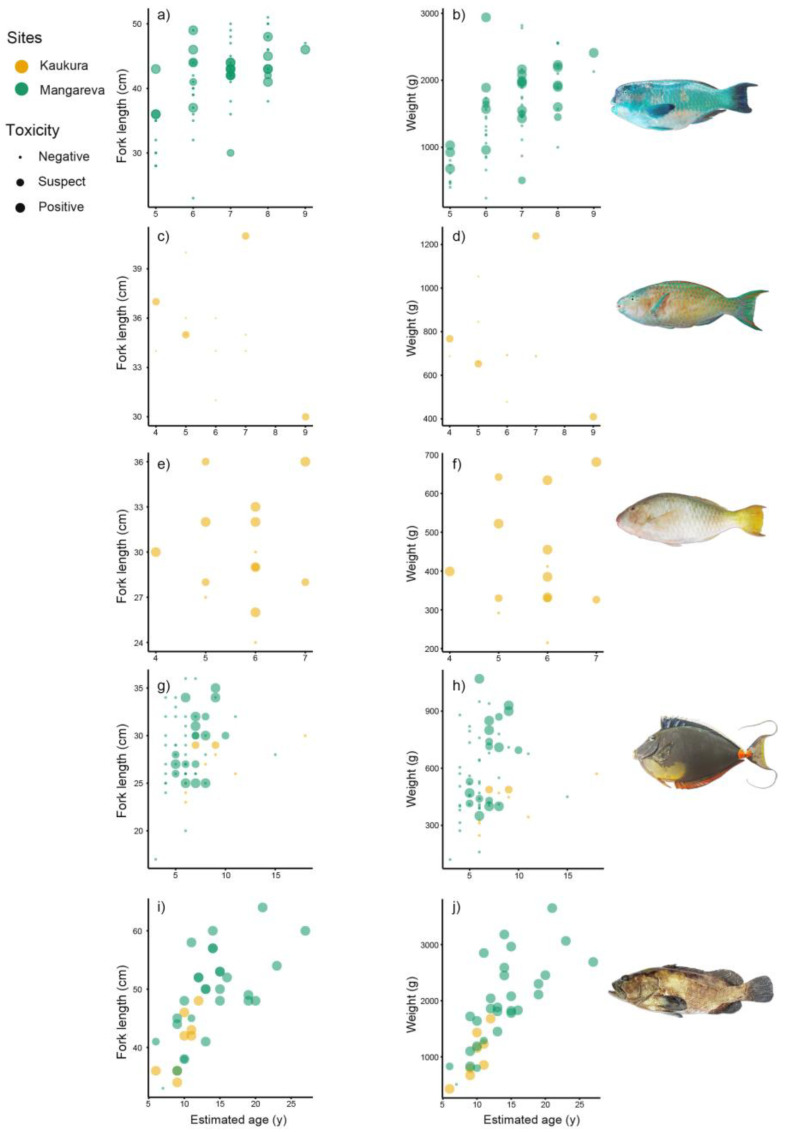
Distribution of fork length (in cm, left column) and weight (in g, right column) according to the specimens’ estimated age and toxic status, in five coral reef fish species caught from Kaukura Atoll and Mangareva Island study sites. (**a**,**b**) *Chlorurus microrhinos* (*n* = 68); (**c**,**d**) *Scarus forsteni* (*n* = 13); (**e**,**f**) *Scarus ghobban* (*n* = 14); (**g**,**h**) *Naso lituratus* (*n* = 68); (**i**,**j**) *Epinephelus polyphekadion* (*n* = 37).

Among the three parrotfish species studied, the age estimation from otolith reading gave a narrow age range of five, three, and four years (Figure 1 and Table S3) despite wider ranges in size and weight of 11, 12, and 28 cm and 830, 466, and 2700 g, for *Scarus forsteni*, *S. ghobban*, and *Chlorurus microrhinos*, respectively.

For *N. lituratus*, an age range of 12 years was observed in the two sampling sites associated with narrower size and weight ranges of 7 cm and 324 g in Kaukura Atoll, and 19 cm and 950 g in Mangareva Island, respectively (Figure 1 and Table S3).

Regarding *E. polyphekadion*, an age range of 21 and six years corresponded to size and weight ranges of 31 cm and 3140 g in Mangareva Island and 14 cm and 1251 g in Kaukura Atoll, respectively (Figure 1 and Table S3). Due to the high heterogeneity observed in the sample size between study sites and the limited number of specimens (10 ≤ *n* < 20) in at least one of the sites (Table S3), comparisons between species or sites were not possible, emphasizing the need for additional sampling efforts in the future to confirm these results. However, these size, weight, and age data are consistent with those previously observed in the East Asia and Pacific regions for the three parrotfish species [39,40,41], the orangespine unicorn fish [42,43], and the marbled grouper [44,45].

The graphical representation of demographic data highlights the marked variability in size and/or weight within a same age group whatever the fish species considered (Figure 1). For example, among the parrot fish species, the six-year-old *C. microrhinos* aged group (*n* = 19) from Mangareva Island showed wide ranges of weight and size of 240–2940 g and 23–49 cm, respectively (Figure 1). In the Acanthuridae, the six-year-old *N. lituratus* aged group (*n* = 16) from Mangareva Island had wide ranges of weight and size of 160–1070 g and 20–36 cm respectively (Figure 1). In the Serranidae, the nine-year-old *E. polyphekadion* aged group (*n* = 3) from Mangareva Island had wide ranges of weight and size of 830–1720 g and 36–45 cm, respectively (Figure 1). Size/weight data are largely influenced by sex-driven growth rate differences, reproductive pattern, spawning season, hermaphroditism, fishing pressure, etc. [39,40,41,42,44,46].

### 2.2. Relationship between Size, Weight, and Age and Fish Ciguatoxicity

Two-way (species × ciguatoxicity) PERMANOVAs revealed significant differences in size and weight, but not in age, between ciguatoxicity groups (Table 1).

**Figure 2:**
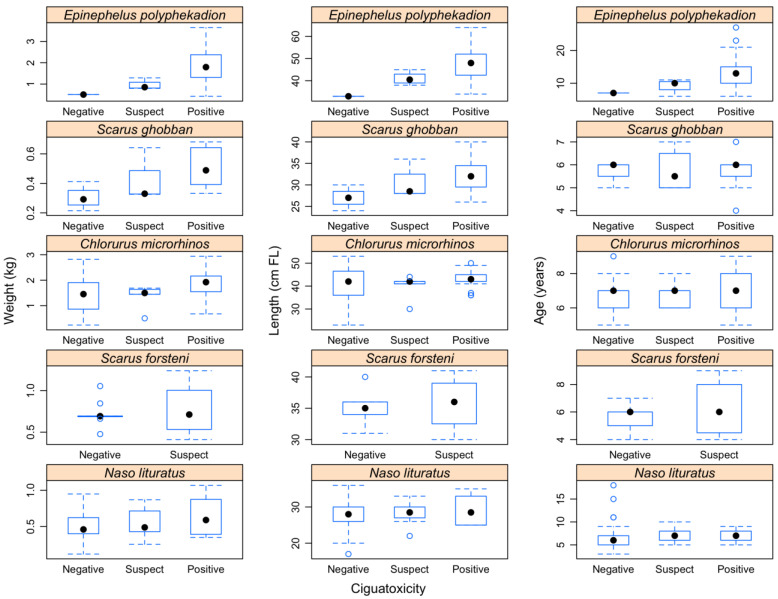
Boxplots between the ciguatoxic status and weight (in kg), fork length (in cm), and estimated age (in years) for each studied fish species *Epinephelus polyphekadion* (*n* = 45), *Scarus ghobban* (*n* = 15), *Chlorurus microrhinos* (*n* = 70), *Scarus forsteni* (*n* = 13), and *Naso lituratus* (*n* = 77) from Mangareva Island and Kaukura Atoll.

Planned comparisons further indicated that fish that tested positive for ciguatoxicity were greater in size and weight than others, with no significant difference between fish that tested negative or suspect (Table S4). The interaction of species × ciguatoxicity was not significant in the PERMANOVAs (Table 1), suggesting that the relationship between size, weight, and ciguatoxicity was consistent across species. Differences in size and weight across ciguatoxicity groups were particularly marked for the grouper *E. polyphekadion*, the parrotfish *S. ghobban* and, to a lesser extent *C. microrhinos*, but were weak for *S. forsteni* and *N. lituratus* (Figure 2). However, the positive group showed high variability in size and weight for all species, indicating that small fish also could be ciguatoxics (Figure 2).

Our results indicate that greater size and weight increase the risk of ciguatoxicity in coral reef fish, but that small fish can still be ciguatoxic due to the large individual variability in size and weight among the positive fish. These paradoxical findings may reconcile contradictory results reported in other studies. A lack of correlation between CTX contents and fish size or weight in Acanthuridae and Scaridae—most notably in *C. microrhinos*—were found in several studies conducted in different islands and atolls of French Polynesia (Table S2 [47]). Consistent with findings reported for parrotfish from the Philippines (Table S2 [48]), these studies suggest that size or weight are not good predictors of fish ciguatoxicity. A negative correlation which suggests a decrease in CTX levels in larger specimens was even observed between CTX contents and fish size in seven reef fish species, most notably *N. unicornis* from the Pacific region [47]. In turn, our findings of higher ciguatoxicity risk in larger fish are also in agreement with those from a previous study showing a positive relationship between ciguatoxicity and the weight of *Seriola dumerilli* from the Atlantic region (Table S2 [5,37]). The existence of a positive correlation between CTX concentration and the total length of another carnivorous fish, moray eels (Muraenidae) from the Pacific region, has also been reported in the literature (Table S2 [18]). Although the effect of size and weight on ciguatoxicity was particularly marked in our study for *E. polyphekadion*, a lack of relationship has also been reported for this species in the Pacific region and in other Serranidae species such as *E. marginatus*, *E. merra*, *E. guttatus*, *Cephalopholis argus,* and *Plectropomus laevis* (Table S2 [18,47,48,49,50,51,52,53]). More widely, a lack of relationship or weak-to-moderate relationships have been reported in several carnivorous fish families from the Southeast Asia and Pacific region (Muraenidae, Lutjanidae, Lethrinidae, and Sphyraenidae), the Caribbean (Scorpionaeidae, Balistidae, Haemulidae, and Carangidae), and the Atlantic (Carangidae and Sphyraenidae) regions (Table S2 [5,18,47,48,49,50,51,52,53,54,55,56,57]). Even more, studies conducted on the same fish species sometimes led to contradictory findings, as is the case for *Caranx latus* from the Caribbean (Table S2 [54,56]). Taken together, all these observations suggest neither the length/weight of individuals, nor the estimated age within a given fish species are good predictors of fish ciguatoxicity. Although it seems correct to state that larger fish pose an increased risk of ciguatoxicity, our study along with many others also indicate that the consumption of small fish still poses serious risks of poisoning. For another grouper, *Variola louti*, a significant yet weak correlation was reported between CTX content and fish size, age, and weight [58]; suggesting that although large size, age, and weight are risk factors, consuming small specimens yet remains risky. These observations call into question the relevance of the regulations adopted in several countries with regards to weight and/or size limits of certain commercially important fish species, such as in the Canary Islands [37], New Caledonia [30], Australia [27], and Hong Kong [31].

### 2.3. Relationship between Fish Ciguatoxicity and Growth Characteristics

**Figure 3:**
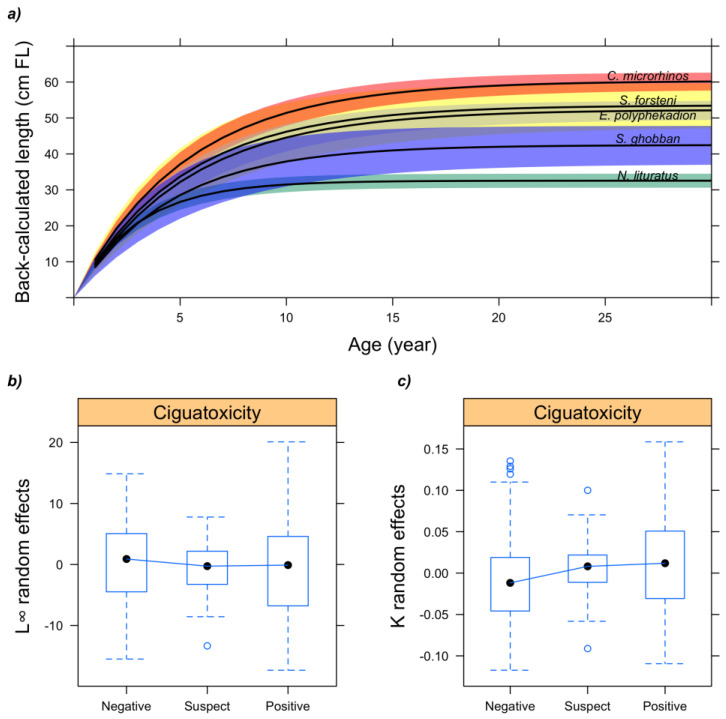
(**a**) Modeled non-linear mixed-effects (NLME) growth trajectories of five coral-reef fish species. Lines represent fixed-effect estimates of the von Bertalanffy growth equation; colored areas represent 95% confidence intervals in estimates for each species. Ciguatoxicity was not included in the NLME growth model since random effects for both (**b**) asymptotic body length (*L*∞) and (**c**) growth rate coefficient (K) showed no trend with ciguatoxicity. See Table 2 for model specification.

The relationship between ciguatoxicity and fish growth characteristics was assessed for all five fish species using non-linear mixed-effects (NLME) modeling of the von Bertalanffy growth equation on length-at-age data back-calculated from otolith radius-at-age data. The model-building strategy for identifying and including covariates in the model showed that the best NLME model only included highly significant random effects and species fixed effects for both growth rate coefficient (K) and asymptotic body length (*L∞*) (Table 2 and Figure 3).

**Table 2:** Non-linear mixed-effects (NLME) model fit of the von Bertalanffy growth equation to length-at-age data back-calculated from otolith analysis for five coral reef fish species. (**a**) Likelihood ratio tests and Akaike and Schwarz information criterion statistics (AIC and BIC, respectively) showing that the best model (in italic) includes significant fixed-effects for species covariate and random-effects on both growth rate coefficient (K) and asymptotic body length (*L∞*). (**b**) Wald-type tests [59] showing significant species effect for both K and *L∞*.

(a)	Model	Fixed Effect	Random Effect	df	AIC	BIC	logLik	Test	L.Ratio	*p*-Value
	1	Intercept	K, *L∞*	6	7440	7472	−3714			
	*2*	*Species K, L∞*	*K, L∞*	14	7247	7323	−3609	1 vs. 2	208	<0.0001
	3	Species K, *L∞*	*L∞*	12	7869	7934	−3922	2 vs. 3	625	<0.0001
	4	Species K, *L∞*	K	12	7863	7928	−3919	2 vs. 4	619	<0.0001
**(b)**		**Fixed Effects**	**numDF**	**denDF**	**F-Value**	***p*-Value**				
		*L∞*.(Intercept)	1	1531	21,467	<0.0001				
		*L∞*.Species	4	1531	139	<0.0001				
		K.(Intercept)	1	1531	1941	<0.0001				
		K.Species	4	1531	37	<0.0001				

Post-hoc comparisons further revealed that *C. microrhinos* had significantly the largest *L∞*, followed by *E. polyphekadion*, *S. ghobban*, and *N. lituratus* (Table S5 and Figure S1). *S. forsteni* asymptotic length was not significantly different from other species, except for *N. lituratus* which showed the smallest *L∞*. Growth rate K was similar among species, except for *N. lituratus* which showed significantly larger K than other species (Figure S1 and Table S5). Individual variations in growth (i.e., random effects of the best NLME growth model) showed no trend with ciguatoxicity, suggesting no relationship between CTX tissue accumulation and the growth characteristics of the five fish species analyzed (Figure 3). The same observations applied to the site and species covariates (Figure S2), indicating that (i) no variation in growth could be evidenced between sites and (ii) inter-species variability in growth was well accounted for by the fixed species effect in the NLME model.

Information regarding the sex of individuals analyzed in this study would have possibly helped better assess the relationships between ciguatoxicity and age and growth since sexual dimorphism in size and growth rate is common among fish [42,44,60]. However, our result also indicates that growth and ciguatoxicity were not linked. Slow-growing individuals (smaller-at-age) or slow-growing species were not more (or less) ciguatoxic than fast-growing ones (larger-at-age). Likewise, species or individuals that could grow to larger sizes were not more (or less) ciguatoxic than others. These results are consistent with those reported on age, size, and weight. Indeed, strong individual variability in growth with no relationship between growth and ciguatoxicity leads to strong variability in age for larger fish, hence explaining why ciguatoxic fish were significantly larger but not older than others. To date, our study is among the first to investigate the relationship between ciguatoxicity and age and growth in coral reef fishes.

### 2.4. Influence of Other Factors on Fish Ciguatoxicity

If, despite increased risk effects for size and weight, the ciguatoxicity cannot be predicted by either size, weight, or age, the question remains about which driver or biological trait could be a good determinant of fish ciguatoxicity and, in an underlying way, could explain it. Several studies have previously examined the potential influence of a variety of other factors (Table S2). Here, the influence of sampling season, certain environmental features of the sampling areas, trophic levels of fish species, and CTX bioaccumulation and depuration kinetics are briefly discussed.

#### 2.4.1. Sampling Season

Season-driven fluctuations of *Gambierdiscus* species abundances and algal CTX fluxes, which in turn might affect CTX levels in marine organism in upper trophic stages have been reported in the literature, although contradictory findings across study regions prevent any definite conclusion [12,61]. In our study, fish were sampled mainly at the start and end of the hot and rainy season, thus it was not surprising that no specific trend could be identified from our data. This is consistent with findings in the Canary Islands where no relationship was observed between occurrence of toxic fish and warm or cold months (Table S2), whereas the frequency of positive reef fish samples seemed to be correlated with the abundance of the toxic benthic dinoflagellates during the dry season in the Philippines (Table S2).

#### 2.4.2. Environmental Features of Sampling Areas

In Kaukura Atoll, *Gambierdiscus* abundance was less than 17 cells g^−1^ of algae against 850 cells g^−1^ of algae in Mangareva Island (data not shown). During the study period (2012–2013), 15 strains of *Gambierdiscus* could be isolated from wild samples from Mangareva Island and established in the laboratory. Using molecular tools, they were identified as *G. australes* (*n* = 2), *G. caribaeus* (*n* = 9), *G. pacificus* (*n* = 1), *G. toxicus* (*n* = 1), and *G. polynesiensis* (*n* = 2). Following additional sampling, this island is regarded as a biodiversity hotspot of *Gambierdiscus* as at least six *Gambierdiscus* species—including *G. polynesiensis*—were identified in wild samples [62,63] and in vitro cultures established from field samplings also allowed to isolate two highly toxic strains of *G. polynesiensis* from Mangareva [64,65]. Among the *G. polynesiensis* strains collected in 2013, one strain—namely, RIK7—was estimated at 3.3 ± 0.2 pg CTX3 eq cell^−1^ by CBA-N2a, and its toxin profile was composed of CTX3B, CTX3C, CTX4A, CTX4B, 2-OH-CTX3C, and two CTX3B/C isomers [64,65] highlighting its high toxic potential. Unfortunately, no data on the toxicity and/or toxin profile of *Gambierdiscus* strains originating from Kaukura Atoll are currently available. The presence of selected, highly toxic species/strains (even if they may not be the numerically dominant ones) in *Gambierdiscus* blooms likely play a prominent role in CP outbreaks and severity [66,67,68]. Previous studies by Yogi et al. [69] and Ikehara et al. [14] also suggest toxin profiles encountered in *Gambierdiscus* spp. blooms likely influence fish ciguatoxicity in ciguatera-prone areas. In particular, the species *G. polynesiensis* is regarded as the dominant producer of CTXs in the food webs in French Polynesian lagoons [64].

#### 2.4.3. Trophic Level

A common belief is that the frequency of CTX occurrence is higher in carnivorous versus herbivorous fish [70]. In our study, the proportion of toxic specimens in Mangareva Island were 30% and 18% for the two herbivores, *C. microrhinos* (*n* = 70) and *N. lituratus* (*n* = 62), respectively, versus 86% for the carnivore *E. polyphekadion* (*n* = 37). A similar trend was observed in Kiribati, where CTXs were detected in 54% and 76% of herbivorous and carnivorous fish, respectively [18]. However, field data from previous studies actually reveal the proportion of toxic specimens do not necessarily increase with the trophic levels but may vary according to the fish family, as observed in French Polynesia, where 100% of the herbivorous Acanthuridae were found positive versus only 47% in Serranidae [47]. This has major implications in terms of risk management considering the strong local dietary preferences observed in some islands where communities tend to prefer eating herbivorous rather than carnivorous species, as is the case in the Pacific region [71,72,73]. Indeed, herbivorous coral reef fish are major contributors of CP events in the Pacific, with Scaridae, Acanthuridae, and Kyphosidae frequently involved in CP cases occurring in the Cook Islands [72] and French Polynesia [17,50,71,74,75].

#### 2.4.4. CTX Bioaccumulation and Depuration Kinetics

The complex metabolic processes involved in toxin assimilation explain the differences in uptake and detoxification capacity observed among fish species [20,76,77,78,79,80,81,82]. Indeed, 2% of the ingested dose were retained in the flesh of *Naso brevirostris* at the end of the experiment [79] compared to 5% in the tissues of *Mugil cephalus* [78]. Unfortunately, such data are not available for the fish species analyzed in the present study. Moreover, fast-growing fish species are expected to contain lower CTX concentrations than a slow-growing species due to somatic dilution [79,83], which may partly explain the absence of a relationship between CTX concentration and total fish length, as growth rates are highly variable between fish families, particularly among reef fish species [84]. Furthermore, in our study, CTXs were measured only in muscle tissues, however, these toxins are known to bioaccumulate differentially in other fish tissues, e.g., in the viscera, liver, kidneys, gall balder, stomach, intestine, gills, eyes, etc. [78,79,80,81,85,86]. Additionally, different uptake rates were observed between CTXs analogs as well as between fish tissues in *Epinephelus coioides* [80]. Regarding CTX depuration, excretion rates (i.e., half-lives) of 143–148, 264, and 900 days were documented in the omnivorous *Lagodon rhomboides* and the carnivorous predatory fishes *Gymnothorax javanicus* and *Lutjanus bohar*, respectively [76,77,81], a result that favors negative relationship between CTX concentration and size or weight. Furthermore, depending on the nature of CTX analogs, half-lives ranging from 4 to 125 days were found for *E. coioides* [80]. Elimination trends also varied according to the fish tissues analyzed, with the highest uptake and elimination rates observed in the liver and the slowest in the muscle [80]. All these findings highlight the diversity of factors likely to influence CTX concentration in fish [82], unfortunately, most of these factors remain poorly documented in coral reef fishes.

### 2.5. Fish Ciguatoxicity versus Epidemiological Data in the Two Study Sites

Toxicity screening by CBA-N2a indicates the proportion of positive fish were 33% (*n* = 51) in Kaukura Atoll versus 60% (*n* = 50) and 28% (*n* = 119) in Mangareva Island in 2012 and 2013, respectively. Given the guidance levels for CTXs established at 0.01 µg equivalents of CTX1B kg^−1^ of fish and the LOQ of the CBA-N2a (0.064 ± 0.016 µg CTX1B eq kg^−1^ flesh), virtually all the fish samples classified as positive in this study will induce CP symptoms among people if consumed. For information, quantitative data are available for two fish specimens from Mangareva—i.e., *Chlorurus microrhinos* (6.63 ± 0.74 ng CTX3C eq ng^−1^) and *Epinephelus merra* (3.37 ± 0.74 ng CTX3C eq ng^−1^)—as determined by CBA-N2a [38]. The high ciguatoxicity of this same *C. microrhinos* specimen was also confirmed by the fluorescent receptor binding assay (7.04 ng CTX3C eq ng^−1^) [87] and by liquid chromatography tandem mass spectrometry which could detect the presence of CTX3B, CTX3C, CTX4A, CTX4B, CTX2, and 51-OH-CTX3C analogs in this fish [88]. These data clearly highlight the high CTX levels that can be found in fish specimens of Mangareva food webs, consistent with the presence of *G. polynesiensis* in this island. Next, to get a better idea of the estimated level of CP risk associated with these fish species in both study sites, the epidemiological data available for 2012 and 2013 were further examined to determine to what extent these species were involved in CP cases [10]. In Kaukura Atoll, Scaridae, Acanthuridae, and Serranidae accounted for 26%, 10.5%, and 5% of CP cases, respectively, while in the Gambier Archipelago, they were involved in 30%, 28%, and 3.5%, respectively, for the period 2012–2013. It should be noted that these data are likely biased by dietary preferences of local populations, as well as avoidance strategies towards fish species considered at risk of ciguatera. By way of example, based on 2012–2013 questionnaires surveys (data not shown), *C. microrhinos*, *N. lituratus,* and *E. polyphekadion* are generally avoided in the Gambier Archipelago. In Kaukura Atoll, *C. microrhinos* and *N. lituratus* are considered safe species, unlike *S. ghobban* and *E. polyphekadion*. The proportion of positive fish in each study site was consistent with the fact that CP risk was indeed much higher in Mangareva than in Kaukura. This spatial stratification of ciguateric risk across islands has also been outlined in previous studies [37,48,69,74]. For instance, in the Canary Islands, the risk of catching a fish containing CTXs is at least twice as high in the eastern than in the western islands, especially in amberjack species [37]. In the Philippines, fish toxin occurrence was also site-specific, with the highest proportion of toxic fish evidenced in sites with dead corals [48].

## 3. Materials and Methods

### 3.1. Study Area

**Figure 4:**
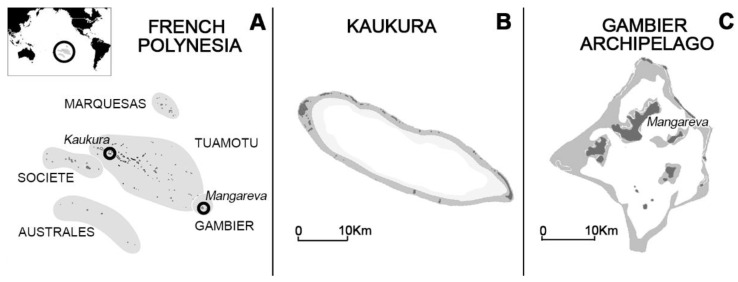
Maps of (**A**) French Polynesia, (**B**) Kaukura Atoll (Tuamotu Archipelago), and (**C**) Mangareva Island (Gambier Archipelago).

Located in the South Pacific, French Polynesia includes about 118 islands, covering an area of 4200 km^2^ scattered over 2,500,000 km^2^ (Figure 4). The territory consists of five archipelagos: Society, Tuamotu, Gambier, Australes, and Marquesas (Figure 4). The 220 fish specimens analyzed in the present study were collected from Kaukura Atoll (Tuamotu Archipelago) and the volcanic Mangareva Island (Gambier Archipelago) (Figure 4).

Kaukura Atoll and Mangareva Island are two contrasted sites with regards to both demographic and anthropogenic pressure. While Kaukura (414 inhabitants based on the last 2017 census) is mainly exploited for lagoon fishing in fishing parks, Mangareva Island (1592 inhabitants) has been a place of intense black pearl farming activities for decades [89]. Based on CP cases incidence rates (IRs) reported through the surveillance network in place in French Polynesia since 2007 [10], Kaukura Atoll is an area with moderate CP risk with a mean IR of 106 ± 70 cases per 10,000 inhabitants prior to 2012, while the Gambier Archipelago has been known as a long-standing CP hotspot since the early 1960s with a mean IR of 494 ± 115 cases per 10,000 inhabitants [63,90,91,92].

### 3.2. Fish Sampling

The five fish species caught from these two sites were selected based on both epidemiological data collected through the above-mentioned surveillance network and on questionnaire surveys conducted with the local populations prior to fish samplings. Fish specimens were sampled by spearfishing in November 2012 in Kaukura and, in May 2012 and February 2013 in Mangareva—i.e., at the beginning and end of the hot, rainy season, respectively—and were distributed among five distinct species: (i) herbivorous steephead parrotfish *Chlorurus microrhinos*, Forsten’s parrotfish *Scarus forsteni*, blue-barred parrotfish *Scarus ghobban* (Scaridae), and orangespine unicornfish *Naso lituratus* (Acanthuridae); and (ii) carnivorous marbled grouper *Epinephelus polyphekadion* (Serranidae) (Table S3). Each specimen was identified at the species level using the guide of Bacchet et al [93], with their size, i.e., the fork length (FL) and total weight measured to the nearest centimeter and gram, respectively (Table S3). The flesh and the head were stored at −20 °C until toxicological and otolith analyses. As with most coral reef fishes, the five studied species are relatively sedentary with home ranges of a few hundred meters to 3–5 km [94]. There is probably a lot of individual variability, but the studied species probably move from reef to reef in the same area (a few km) but cannot move from island to island.

### 3.3. CTXs Chemical Extraction

After homogenization of the whole flesh, CTXs were extracted from 10 g flesh portions following the protocol from Darius et al. [95]. Briefly, each 10 g fish specimen was extracted twice in methanol (MeOH) and twice in aqueous methanol (MeOH/H_2_O) 50/50 for herbivores, or twice in acetone (C_3_H_6_O) for carnivores, under sonication for 4 h. After one night at −20 °C, the crude extracts were centrifuged, and the supernatants were pooled and dried under vacuum. The resulting crude extract was further partitioned between dichloromethane (CH_2_Cl_2_) and MeOH/H_2_O 60/40 twice. The resulting CH_2_Cl_2_ phase was dried under vacuum and further defatted by a second solvent partition using cyclohexane (C_6_H_12_) and MeOH/H_2_O 80/20 (=liposoluble fraction, LF). The MeOH/H_2_O 80/20 LF was then evaporated and further purified on Sep-Pak C18 cartridges (360 mg sorbent per cartridge; Waters^®^, Saint-Quentin, France). The columns were preconditioned with MeOH/H_2_O 70/30 before loading extracts, washed with MeOH/H_2_O 70/30, and eluted successively with MeOH/H_2_O 90/10 and pure methanol, leading to three distinct liposoluble fractions: LF70/30, LF90/10, and LF100. All these fractions were then dried in a SpeedVac concentrator, weighed, and stored at +4 °C. As the majority of CTXs are eluted in the LF90/10 [50,95,96], only this latter fraction was considered to search for the presence of CTXs. The LF90/10 extract was resuspended in pure methanol giving a sample stock solution at a concentration of 10 µg of dry extract µL^−1^.

### 3.4. Neuroblastoma Cell-Based Assay (CBA-N2a)

The presence of composite CTX-like toxicity of fish samples was investigated using the neuroblastoma cell-based assay (CBA-N2a) following the recently optimized protocol described in Viallon et al. [38]. The LF90/10 chemical extract was used to detect an activation of voltage-gated sodium channels specific to the mode of action of CTXs characterized by no cytotoxic effects in the absence of ouabain (O) and veratridine (V) treatment (OV− condition) versus a reduction in cell viability when tested under non-destructive OV treatment (OV+ condition). To establish the ciguatoxic status of fish, a qualitative screening was performed by testing a single extract concentration set at the maximum concentrations of dry extract (MCE) that did not induce unspecific cytotoxic effects in neuroblastoma cells (N2a) (MCE established at 10,000 pg of dry extract µL^−1^) in OV− and OV+ conditions (final concentrations of O/V were between 80/8 and 100/10 µM) [38]. Practically, each stock solution was diluted (1:50 in 2% fetal bovine serum (FBS) culture medium) then 10 µL was directly added to 200 µL of OV− and OV+ treatments (triplicate wells), in one or two experiments. In each microplate, controls in OV− and OV+ conditions—namely, COV− and COV+ controls—were implemented by adding 10 µl of culture medium to check the non-destructive effect of the OV treatment in the absence of samples. In addition, quality check controls (QC) in OV− and OV+ conditions—namely, QCOV− and QCOV+—were also undertaken to check for the specific detection of VGSC activators (no cytotoxicity in OV- condition and cytotoxicity in OV+ condition), using a pure VGSC toxin, i.e., CTX3C (ILM bank of standard) added in triplicate wells as described in Viallon et al. [38]. In this study, to allow easier comparison of our data with the US-FDA guidance level, the LOD and LOQ values originally determined for fish matrices and expressed in µg CTX3C eq kg^−1^ in Viallon et al. [38] were converted into µg CTX1B eq kg^−1^, as CTX3C and CTX1B displayed similar cytotoxic effects on neuroblastoma cells in CBA-N2a [95,97]. As detailed in the protocol guide presented in the supplementary materials of Viallon et al. [38], the absorbance data in both OV− and OV+ conditions obtained for each fish sample following this screening step were monitored and further transformed into viability percentages (%) relative to COV− and COV+ controls, respectively. After checking that those viability percentages in OV− condition were similar (100%) to that of COV- controls, the ciguatoxic status of fish samples was classified into three categories based on the composite CTX-like toxicity detected in fish extracts in OV+ condition [38]. These fish samples were labeled:

—‘negative’ when N2a viability percentages were >80%, i.e., the CTX concentrations (if any) are below the limit of detection (LOD) of the CBA-N2a established at LOD = 0.031 ± 0.008 µg CTX1B eq kg^−1^ flesh. These specimens are considered nontoxic if consumed.

—‘suspect’ when N2a viability percentages were between 20% and 80%, i.e., they could be either free of CTXs or may contain only traces of CTXs between the LOD and the limit of quantification (LOQ) of the CBA-N2a established at LOQ = 0.064 ± 0.016 µg CTX1B eq kg^−1^ flesh.

—‘positive’ when N2a viability percentages were <20%, i.e., they contained CTX concentrations ≥ LOQ. Based on the guidance levels for Pacific CTXs of the US FDA [21], these samples are likely to induce CP symptoms among people if consumed, and thus regarded as toxic. 

### 3.5. Age Estimation

Otoliths are paired calcified structures located in the inner ear of fish. They constitute natural recorders displaying yearly—and for younger individuals, daily—patterns of growth that are used to estimate fish age and growth [98]. Here, sagittal otoliths were removed from each specimen, cleaned, and stored dry. Otolith analyses were performed following the protocol of [99]. For each species, one sagittae per individual was embedded in epoxy resin (Araldite^®^, 2020, Huntsman, Basel, Switzerland) and when dry, transverse thin sections including otolith cores were prepared using a precision low speed saw (Buehler^®^, Isomet 1000, Leinfelden-Echterdingen, Germany). The transverse sections were polished using sandpapers with increasing fine grains (from 800 to 2000 grains.cm^−2^) and refined with abrasive lapping films (from 9 to 0.3 μm) to expose the nucleus of the otolith. Then, all transverse sections were decalcified with a drop of EDTA (ethylenediaminetetraacetic acid 5%) for 1 min 30 sec, allowing highlighting the relief of the growth increments. Once rinsed, transverse sections were colored for 2 min with toluidine blue (1%) to improve otolith reading. The colorant binds to the otolith protein matrix and increases the contrast between translucent and opaque growth bands. Annual increments were observed under a microscope and when they were not clearly visible, the polishing step was repeated [100]. When yearly increments were clearly visible, transversal sections were photographed and examined using the software ImageJ [101]. The counting of the annual increments was carried out twice by two independent readers to estimate the age of each fish. In order to model their growth, a measure of the distance between the nucleus and each annual increment, and between the nucleus and the edge of the otolith (considered as the last annual increment if it was colored) was carried out using ImageJ.

### 3.6. Modeling Fish Growth

Growth back-calculation models enable the inference of body length at ages prior to capture and to reconstruct fish growth trajectories from birth to capture [102]. All models assume that there is a relationship between otolith growth and fish somatic growth. In this study, fish length-at-age was back-calculated from otolith radius-at-age using the Dahl-Lea back-calculation model [103] in which a proportional relationship between otolith radius and fish length is assumed as

(1) $$Lt=\left(\frac{Rt}{Rc}\right)Lc$$

with *Rt* and *Lt:* otolith radius and fish length at age *t*, *Rc* and *Lc:* otolith radius and fish length at capture. 

Fish growth trajectories were modeled using the von Bertalanffy growth model (VBGM), which assumes that growth is the net result of two opposite metabolic processes: anabolism, the production of body substances; and catabolism, the consumption of body substances. The VBGM is the most widely used growth model for fish [104], and is given by the equation [99,105]

(2) $$Lt=L\infty \left(1-e^{-K\left(t-t0\right)}\right)$$

with *Lt*: body length at age *t*, *L∞*: asymptotic body length, *K*: growth rate coefficient (unit is time^−1^), *t*0: theoretical age when length is zero. Note that *t*0 has little biological meaning and was set to zero in this study. 

VBGM growth coefficients *K* and *L∞* were estimated from back-calculated length-at-age dataset using non-linear mixed-effects (NLME) models [59]. NLME are especially appropriate for the modeling of longitudinal, auto-correlated, and invariably unbalanced datasets such as the back-calculated dataset of length-at-age [98]. The random effects in an NLME model account for variation in the parameters among individuals, while the fixed effects are parameter values at population or covariate level.

### 3.7. Covariates of Ciguatoxicity

Toxicity analyses were performed on 220 fish specimens, but the age could be estimated for only 200 of them (90.9%, Table S3). For the 20 remaining individuals, otoliths were either lost or broken during dissections or displayed annual marks were not interpretable for various reasons. From these 200 fish specimens, the relationship between ciguatoxicity and individual size, weight, and age was assessed using two-way (species × ciguatoxicity) permutational multivariate analysis of variance (PERMANOVA). The PERMANOVAs were fitted using the lmPerm R package setting perm = “Prob”, maxIter = 10^7^ and Ca = 0.00001 in the lmp function in order to stabilize permutational probabilities.

The relationship between ciguatoxicity and von Bertalanffy growth coefficients was explored using the NLME model-building strategy defined by Pinheiro [106] for identifying and including covariates in an NLME model. Briefly, the strategy consists in first fitting an NLME model without covariate. Second, estimated random effects were plotted against available covariates. Third, covariates showing a trend with random effects were incorporated in the model with resulting estimated fixed effects being tested for significance. Here, we explored the effects of covariate ciguatoxicity, site, and species. All analyses were performed with R v4.0.2 [107]. The NLME were fitted using the NLME R package [108].

## 4. Conclusions

Our results showed that fish size, weight, estimated age, and growth, are not reliable determinants of fish ciguatoxicity, although size and weight do involve an increased risk of ciguatoxicity. This also applies to the trophic level, fishing site, season, or lipid contents as highlighted in previous studies. Although, according to the food chain theory, the risk of finding CTXs is higher in larger, aged fishes, the current study and others clearly showed smaller fishes can be as toxic as or even more toxic than larger congeners. Adding to the complexity is the fact that, for a given species, large regional differences can sometimes be observed with regards to the occurrence of CTXs in fish tissues [69,109,110]. Moreover, the complex bioaccumulation and depuration processes which remain poorly documented in most coral reef fish species certainly have a significant impact on the CTX-like content measured at a given time in fish samples. Therefore, programs aiming at predicting the ciguatoxicity of fish samples cannot be generalized at a global scale but should be designed by region partly because of the strong local influence of *Gambierdiscus* species occurrence and distribution [109].

Given these considerations, it is concluded that ciguatera risk management programs based solely on fish biological characteristics can be misleading and may possibly lead to a false sense of security, which is damaging when dealing with food safety issues. For the moment, conducting large-scale random toxicity surveys in seafood organisms in order to evaluate the prevalence of ciguatoxic specimens in CP-prone areas—as is already the case in some localities in the Atlantic, Caribbean, and Pacific regions [5,18,37,50,111,112]—would appear to be a much more reliable strategy. However, establishing random fish testing surveys on a regular basis to prevent outbreaks is beyond the capacity of most CP-endemic island states. As an alternative, an early warning approach based on the field monitoring of *Gambierdiscus* species known to be major contributors to CTX flux in CP-prone regions (e.g., *G. polynesiensis* in the Pacific, *G. excentricus* in the Caribbean) can facilitate the identification of areas where CP risk is the greatest [64,113].

For another plan, the current lack of traceability of fish products from harvest to retail outlets is another important issue in CP outbreak management, in that it prevents tracing tainted products back to their toxic source. Accurate labeling of the origin of seafood products in distribution channels is thus a regulatory measure that needs to be enforced as a priority to help minimize the health impacts but also potential economic losses in case of a major toxic event [109].

## Figures and Tables

**Table 1:** Two-way (species × ciguatoxicity) permutational multivariate analysis of variance (PERMANOVAs) of size (fork length), weight, and age of five coral reef fish species (*Epinephelus polyphekadion*, *Scarus ghobban*, *Chlorurus microrhinos*, *Scarus forsteni*, *Naso lituratus*) and three levels of ciguatoxicity (negative, suspect, positive). Significance codes: <0.001 ***, <0.01 **, <0.05 *.

Response	Factor	DF	Sum Sq	Mean Sq	Iter	*p*-Value
Size	Species	4	3852.3	963.08	10^7^	<2 × 10^−16^ ***
	Ciguatoxicity	2	234.2	117.09	10^7^	0.01639 *
	Species:Ciguatoxicity	7	258.6	36.95	10^7^	0.24569
	Residuals	206	5835.8	28.33		
Weight	Species	4	23,076,135	5,769,034	10^7^	<2 × 10^−16^ ***
	Ciguatoxicity	2	2,143,875	1,071,937	10^7^	0.01918 *
	Species:Ciguatoxicity	7	2,642,863	377,552	10^7^	0.19061
	Residuals	206	54,247,969	263,340		
Age	Species	4	77.17	19.2921	10^7^	0.0406 *
	Ciguatoxicity	2	28.73	14.3655	10^7^	0.1126
	Species:Ciguatoxicity	7	77.95	11.1362	10^7^	0.1247
	Residuals	2	28.73	14.3655		0.1126

## Data Availability

Not applicable.

## Supplementary Materials

The following supporting information can be downloaded at: https://www.mdpi.com/article/10.3390/md20040251/s1, Table S1: Occurrence of ciguatoxins (CTXs) analogs in marine food webs. Only formal identifications of CTXs by chemical methods (i.e., liquid chromatography tandem mass spectrometry, LC-MS/MS) were considered; Table S2: Relationship between biological or environmental factors and the CTX concentration or ciguatoxic status of fish samples. Only studies reporting statistical results based on a number of samples with *n* ≥ 20 both by species (or families) and by factors were considered; Table S3: Number of specimens analyzed per species and site for their ciguatoxic status (N_tox_), size (cm, fork length), weight (g), and age (y) estimation (N_age_); Table S4: Planned comparisons of ciguatoxicity levels in two-way (species × ciguatoxicity) permutational multivariate analysis of variance (PERMANOVAs) of size (fork length) and weight of five coral reef fish species (*Epinephelus polyphekadion*, *Scarus ghobban*, *Chlorurus microrhinos*, *Scarus forsteni*, *Naso lituratus*) and three levels of ciguatoxicity (negative, suspect, positive); Table S5: Non-linear mixed-effects (NLME) estimates and 95% confidence intervals (CI) of von Bertalanffy growth coefficients in five coral reef fish species; Figure S1: Post-hoc comparisons of species fixed-effects in the best non-linear mixed-effects (NLME) modeling of growth trajectories using the von Bertalanffy growth equation. (A) Asymptotic body length (*L*∞). (B) Growth rate coefficient (K). Grey boxes represent 95% confidence intervals in species fixed-effect estimates. Non-overlapping arrows indicate significant differences in species estimates with Tukey post-hoc tests; Figure S2: Distribution among ciguatoxicity classes (A,B), sites (C,D), and species (E,F) of individual variation in growth, i.e., random effects of non-linear mixed-effects (NLME) modeling of growth for both von Bertalanffy growth rate coefficient (K) and asymptotic body length (*L*∞). The plots indicate no detectable relationship between growth and ciguatoxicity or site, and that species effect was well accounted for by the model. See Table 2 for model specification. References [114,115,116,117,118,119,120,121,122,123,124,125,126,127,128,129,130,131,132,133,134,135,136,137,138,139,140,141,142,143,144,145,146,147,148,149,150,151,152,153,154,155,156,157,158,159,160,161,162,163,164] are cited in the supplementary materials.
