# Supplementary material for: Evaluating Age and Growth Relationship to Ciguatoxicity in Five Coral Reef Fish Species from French Polynesia
Source: Mar Drugs. 2022 Apr 1;20(4):251. doi: 10.3390/md20040251 (PMC9027493; doi:10.3390/md20040251)
Supplement: Supplementary file 1 [file marinedrugs-20-00251-s001.zip › marinedrugs-1651432-supplementary.pdf]

marine drugs

MDPI

Supplementary Materials: Evaluating Age and Growth Relationship to Ciguatoxicity in Five Coral Reef Fish Species from French Polynesia

Hélène Taiana Darius [1,*], Christelle Paillon [2], Gérard Mou-Tham [2], André Ung [1], Philippe Cruchet [1], Taina Revel [1], Jérôme Viallon [1], Laurent Vigliola [2], Dominique Ponton [3], and Mireille Chinain [1]

1 Institut Louis Malardé (ILM), Laboratory of Marine Biotoxins, UMR 241-EIO (IFREMER, ILM, IRD, Université de Polynésie Française), P.O. Box 30, 98713 Papeete, Tahiti, French Polynesia; aung@ilm.pf (A.U.); pcruchet@ilm.pf (P.C.); trevel@ilm.pf (T.R.); jviallon@ilm.pf (J.V.), mchinain@ilm.pf (M.C.)
2 ENTROPIE, IRD-Université de La Réunion-CNRS-Université de la Nouvelle-Calédonie-IFREMER, Labex Corail, 98848 Nouméa, New Caledonia, France; christelle.paillon@gmail.com (C.P.); moutham.g@gmail.com (G.M-T.); laurent.vigliola@ird.fr (L.V.)
3 ENTROPIE, IRD-Université de La Réunion-CNRS-Université de la Nouvelle-Calédonie-IFREMER, c/o Institut Halieutique et des Sciences Marines (IH.SM), Université de Toliara, Rue Dr. Rabesandratana, BP 141, 601 TOLIARA – Madagascar; dominique.ponton@ird.fr
* Correspondence: tdarius@ilm.pf ; Tel.: +689-40-416-484

Table S1. Occurrence of ciguatoxins (CTXs) analogs in marine food webs. Only formal identifications of CTXs by chemical methods (i.e., liquid chromatography tandem mass spectrometry, LC-MS/MS) were considered.

**Trophic stage**	**Pacific CTXs**	**Caribbean CTXs**	**Indian CTXs**
Gambierdiscus	CTX3C, CTX3B, CTX4A, CTX4B, 2-hydroxyCTX3C, 51-hydroxyCTX3C, 54-deoxy-CTX1B, 52-*epi*-54-deoxy-CTX1B, M-*seco*-CTX3C, M-*seco*-CTX3Cmethyl acetal, M-*seco*-CTX4A/B, 2-OH-CTX3C, CTX3B/C isomers 2-3 [64,65,88,114–127]	ND*	ND
Fish	CTX1B, CTX1A, CTX4A, CTX4B, CTX4C, 52-*epi*-54-deoxyCTX1B, 54-deoxyCTX1B, 7-oxo-CTX1B, 7-hydroxyCTX1B, 4-hydroxy-7-oxoCTX1B, 54-deoxy-50-hydroxyCTX1B, 51-hydroxyCTX3C, 2,3-dihydro-2,3-dihydroxyCTX3C, 2,3-dihydro-2-hydroxyCTX3C, 2,3-dihydro-51-hydroxy-2-oxoCTX3C, 2,3-dihydro-2,3,51-trihydroxy-CTX3C, A-*seco*-2,3-dihydro-51-hydroxyCTX3C, M-*seco*-CTX4A/4B, M-*seco*-CTX3C, CTX3C, CTX3B [17,18,32–34,50,51,69,86,88,110,114,117–119,121,127–134,136–146]	C-CTX1, C-CTX-2, C-CTX3, C-CTX4, C-CTX5-12** [2,3,36,52,53,56,87,111,135,147–159]	I-CTX-1-6** [160,161]
Marine invertebrates	CTX3C, CTX3B, CTX4A, CTX4B, 51-hydroxyCTX3C, CTX1B, M-*seco*-CTX3C, 54-deoxyCTX1B, 52-*epi*-54-deoxyCTX1B [18,88,95,162]	ND	ND
Sharks	ND	ND	I-CTX1-6** [163]

*ND : not documented.**Structure not elucidated.

Table S2. Relationship between biological or environmental factors and the CTX concentration or ciguatoxic status of fish samples. Only studies reporting statistical results based on a number of samples with n ≥ 20 both by species (or families) and by factors were considered.

	Size	Weight	Trophic level	Lipid content	Fishing site	Season
Absence or weak to moderate relationship	Pacific Region<br>Acanthuridae<br>*Acanthurus leucopareius, Ctenochaetus striatus, Naso unicornis*<br>Scaridae<br>*Chlorurus microrhinos*[a], *Leptoscarus vaigiensis, Scarus altipinnis, S. rubroviolaceus*<br>Kyphosidae<br>*Kyphosus cinerascens*<br>Serranidae<br>*Cephalopholis argus, Epinephelus polyphekadion*[a], *Plectropomus laevis*<br>[18,47,50,51]	Acanthuridae<br>*Acanthurus leucopareius, Ctenochaetus striatus, Naso unicornis*<br>Scaridae<br>*Chlorurus microrhinos*[a], *Leptoscarus vaigiensis, Scarus altipinnis, S. rubroviolaceus*<br>Kyphodiae<br>*Kyphosus cinerascens*<br>Serranidae<br>*Cephalopholis argus, Epinephelus polyphekadion*[a], *Plectropomus laevis*<br>Muraenidae<br>*Gymnothorax flavimarginatus*<br>[18,47,49–51]	Herbivores<br>Scaridae,<br>Acanthuridae<br>Omnivores<br>Kyphosidae<br>Carnivores<br>Lutjanidae,<br>Serranidae,<br>[47]	Serranidae<br>*Cephalopholis argus*<br>Muraenidae<br>*Gymnothorax flavimarginatus, Gymnothorax javanicus*<br>[18]	ND	ND
	Asia region<br>ND	Scaridae<br>*Scarus quoyi*<br>Siganidae<br>*Siganus guttatus*<br>Lutjanidae<br>*Lutjanus campechanus*<br>Lethrinidae<br>*Lethrinus letjan*<br>Serranidae<br>*Epinephelus merra*<br>Sphyraenidae<br>*Sphyraena barracuda*<br>[48][c]	ND	ND	ND	Scaridae<br>*Scarus quoyi*<br>Siganidae<br>*Siganus guttatus*<br>Lutjanidae<br>*Lutjanus campechanus*<br>Lethrinidae<br>*Lethrinus letjan*<br>Serranidae<br>*Epinephelus merra*<br>Sphyraenidae<br>*Sphyraena barracuda*

						[48][c]
	Caribbean region<br>Scorpionaeidae<br>*Pterois sp.*<br>Balistidae<br>*Balistes vetula*<br>Serranidae<br>*Epinephelus guttattus*<br>Haemulidae<br>*Haemulon plumierii*<br>[52,57]	Scorpionidae<br>*Pterois sp*<br>Balistidae<br>*Balistes vetula*<br>Carangidae<br>*Caranx bartholomaei*<br>Serranidae<br>*Epinephelus guttattus*<br>Haemulidae<br>*Haemulon plumierii*<br>[52,54,57]	ND	ND	ND	ND
	Atlantic region<br>Sphyraenidae<br>*Sphyraena barracuda*<br>[55]	Carangidae<br>*Caranx latus*<br>Seriola fasciata<br>Serranidae<br>*Epinephelus marginatus*<br>[5,37,53,56][c]	ND	ND	ND	Carangidae<br>*Seriola dumerilli,*<br>*S. rivoliana*<br>[5,37][c]
Significant relationship	Pacific region<br>Lutjanidae<br>*Lutjanus bohar*<br>Muraenidae<br>*Gymnothorax flavimarginatus,*<br>*Gymnothorax javanicus*<br>[18,47]	Muraenidae<br>*Gymnothorax javanicus*<br>[18]	ND	ND	Serranidae<br>*Cephalopholis argus*<br>[164][c]	ND
	Asia region<br>ND	ND	ND	ND	ND	Scaridae<br>*Scarus quoyi*<br>Siganidae<br>*Siganus guttatus*<br>Lutjanidae<br>*Lutjanus campechanus*

						Lethrinidae<br>*Lethrinus letjan*<br>Serranidae<br>*Epinephelus merra*<br>Sphyraenidae<br>*Sphyraena barracuda*<br>[48]c
	Caribbean region<br>ND	Carangidae<br>*Caranx latus*<br>[54]	ND	ND	ND	ND
	Atlantic region<br>ND	Carangidae<br>*Seriola dumerilli, S. rivoliana*<br>[5,37][c]	ND	ND	Carangidae<br>*Seriola dumerilli, Seriola rivoliana*<br>Serranidae<br>*Epinephelus marginatus*<br>[5,37,53][c]	ND

[a]Same fish species as the one selected in our study. [b]ND: Not documented. [c]Ciguatoxin data were established according to ciguatoxicity status from percentages of cell viability using neuroblastoma cell-based assay (CBA-N2a) or symptoms observed in mice using mouse bioassay (MBA).

Table S3. Number of specimens analyzed per species and site for their ciguatoxic status (N_{tox}), size (cm, fork length), weight (g), and age (y) estimation (N_{age}).

		Kaukura					Mangareva					Total	
Species	Ciguatoxic status*	N_{tox}	N_{age}	Size (cm)	Weight (g)	Age (y)	N_{tox}	N_{age}	Size (cm)	Weight (g)	Age (y)	N_{tox}	N_{age}
C. microrhinos	Negative						44	43	23–51	240–2820	5–9	44	43
	Suspect						5	5	30–44	505–1690	6–8	5	5
	Positive						21	20	36–49	680–2940	5–9	21	20
Total Cmic		0	0	0	0		70	68	70	70	68	70	68
S. forsteni	Negative	9	9	31–40	477–1054	4–7						9	9
	Suspect	4	4	30–41	410–1240	4–9						4	4
	Positive	0	0									0	0
Total Sfor		13	13	13	13	13	0	0	0	0		13	13
S. ghobban	Negative	3	3	24–30	215–412	5–6						3	3
	Suspect	4	4	28–36	326–642	5–7						4	4
	Positive	8	7	26–36	332–681	5–7						8	7
Total Sgho		15	14	15	15	14	0	0	0	0		15	14
N. lituratus	Negative	9	7	23–30	247–571	6–18	42	41	17–36	120–950	3–15	51	48
	Suspect	5	2	29	488	7–9	9	8	26–32	415–870	5–10	14	10
	Positive	1	0				11	10	25–35	350–1070	5–9	12	10
Total Nlit		15	9	15	15	9	62	59	62	62	59	77	68
E. polyphekadion	Negative	0					1	1	33	510	7	1	1
	Suspect	0					4	3	38–45	800–1290	6–11	4	3
	Positive	8	8	34–48	429–1680	6–12	32	25	36–64	830–3650	9–27	40	33
Total Epol		8	8	8	8		37	29	37	37	29	45	37
Total		51	44	51	51	44	169	156	169	169	156	220	200

*The ciguatoxic status was established according to the neuroblastoma cell-based assay (CBA-N2a) results (see § 3.4). Of note, all these samples were also tested using the radioactive receptor binding assay (rRBA) (data not shown).

Table S4. Planned comparisons of ciguatoxicity levels in two-way (species × ciguatoxicity) permutational multivariate analysis of variance (PERMANOVAs) of size (fork length) and weight of five coral reef fish species (*Epinephelus polyphekadion, Scarus ghobban, Chlorurus microrhinos, Scarus forsteni, Naso lituratus*) and three levels of ciguatoxicity (negative, suspect, positive).

Response	Comparison	Factor	DF	Sum Sq	Mean Sq	Iter	*p*-value
Size	Negative - Suspect	Species	4	2134.8	533.71	10^7	$<2 \times 10^{-16}$***
		Ciguatoxicity	1	50.6	50.64	10^7	0.161
		Species:Ciguatoxicity	4	68.9	17.23	10^7	0.6155
		Residuals	129	3268.9	25.34		
	Negative - Positive	Species	4	4662.6	1165.64	10^7	$<2.2 \times 10^{-16}$***
		Ciguatoxicity	1	337.5	337.52	10^7	0.0005101***
		Species:Ciguatoxicity	3	188.1	62.69	10^7	0.0988557
		Residuals	180	5482.1	30.46		
	Suspect - Positive	Species	4	3170.8	792.7	10^7	$<2 \times 10^{-16}$***
		Ciguatoxicity	1	163.7	163.72	10^7	0.01774*
		Species:Ciguatoxicity	3	93.2	31.05	10^7	0.356
		Residuals	103	2920.6	28.36		
Weight	Negative - Suspect	Species	4	11071231	2767808	10^7	1×10^{-7}***
		Ciguatoxicity	1	107299	107299	10^7	0.4483
		Species:Ciguatoxicity	4	234158	58540	10^7	0.87
		Residuals	129	25432305	197150		
	Negative - Positive	Species	4	31747716	7936929	10^7	$<2.2 \times 10^{-16}$***
		Ciguatoxicity	1	2403490	2403490	10^7	0.004543**
		Species:Ciguatoxicity	3	1358031	452677	10^7	0.193043
		Residuals	180	52274663	290415		
	Suspect - Positive	Species	4	15197253	3799313	10^7	$<2.2 \times 10^{-16}$***
		Ciguatoxicity	1	2206487	2206487	10^7	0.007699**
		Species:Ciguatoxicity	3	1694638	564879	10^7	0.133591
		Residuals	103	30788970	298922		

No planned comparisons were made for age since this response variable showed no significant effects (see Table 1 in main text).
Significance codes: <0.001 ***, <0.01 **, <0.05 *.

Table S5. Non-linear mixed-effects (NLME) estimates and 95% confidence intervals (CI) of von Bertalanffy growth coefficients in five coral reef fish species

Species	Fixed-effects	Lower 95% CI*	Estimate	Upper 95% CI
Chlorurus microrhinos	L_∞	57.9	60.3	62.7
Scarus forsteni	L_∞	47.5	53.5	59.6
Scarus ghobban	L_∞	37.1	42.5	47.8
Naso lituratus	L_∞	30.6	32.5	34.5
Epinephelus polyphekadion	L_∞	49.7	52.3	54.8
Chlorurus microrhinos	K	0.174	0.191	0.207
Scarus forsteni	K	0.157	0.198	0.239
Scarus ghobban	K	0.179	0.223	0.267
Naso lituratus	K	0.321	0.342	0.363
Epinephelus polyphekadion	K	0.170	0.191	0.212

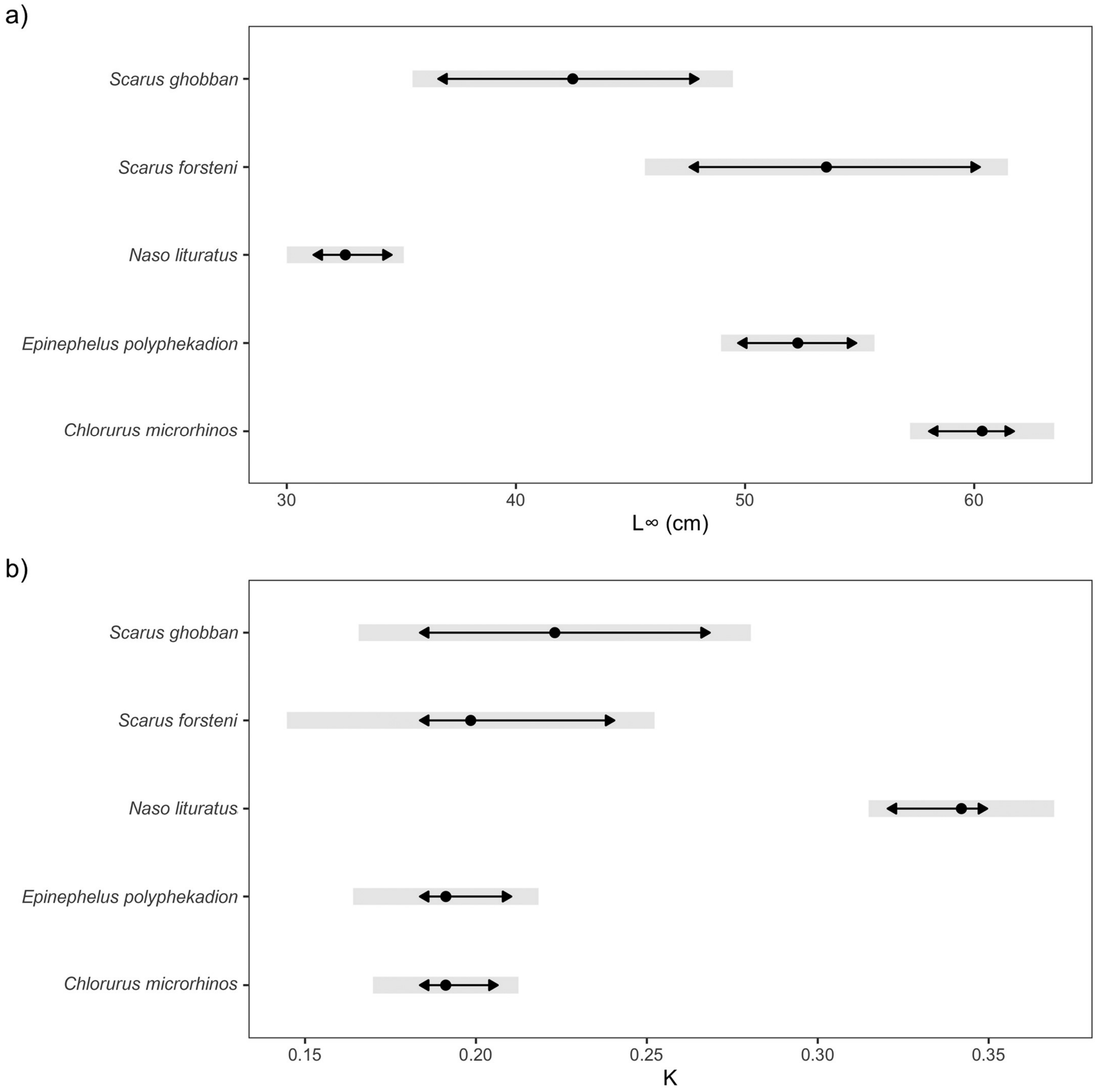

Figure S1. Post-hoc comparisons of species fixed-effects in the best non-linear mixed-effects (NLME) modeling of growth trajectories using the von Bertalanffy growth equation. (**A**) Asymptotic body length (L∞). (**B**) Growth rate coefficient (K). Grey boxes represent 95% confidence intervals in species fixed-effect estimates. Non-overlapping arrows indicate significant differences in species estimates with Tukey post-hoc tests.

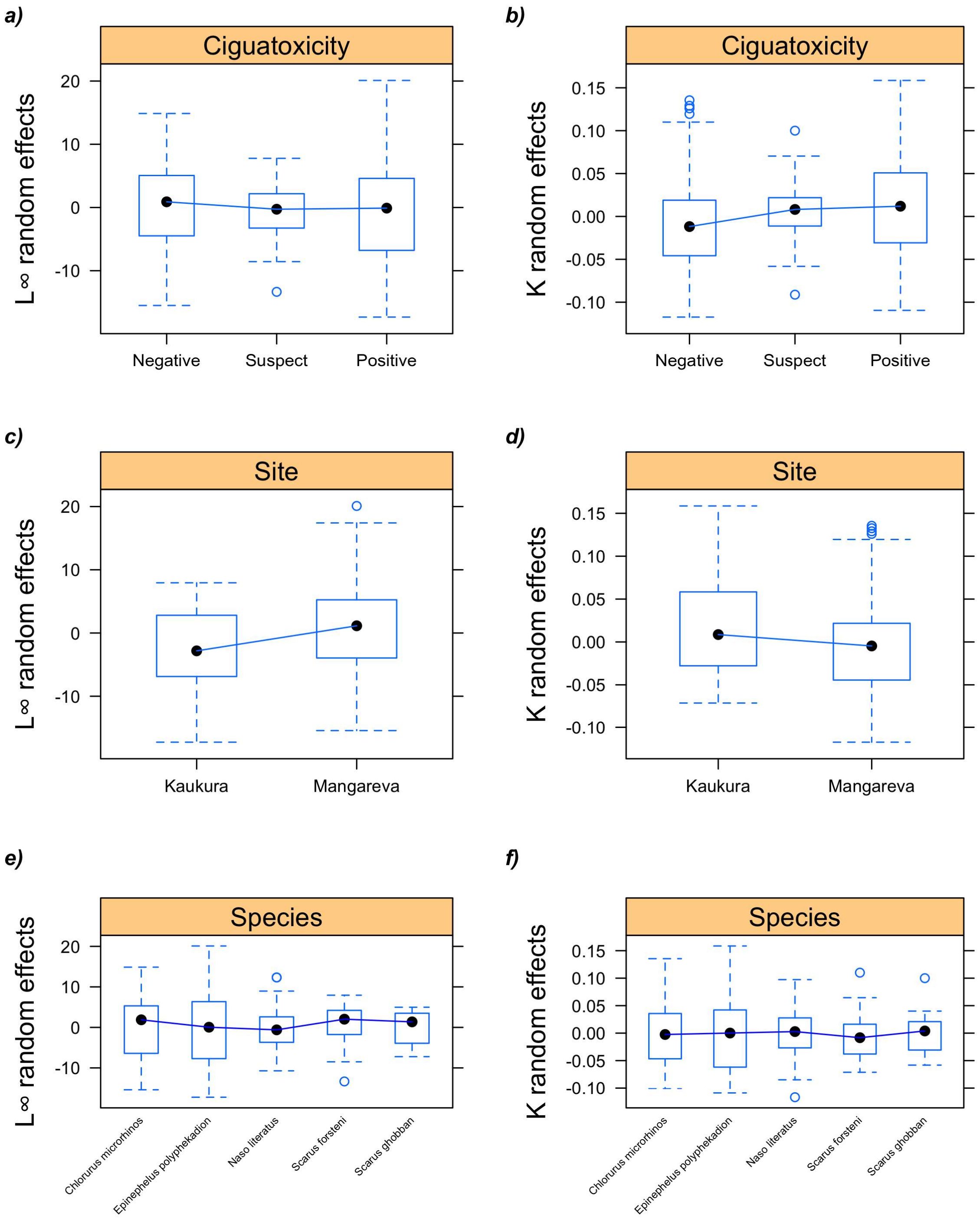

Figure S2. Distribution among ciguatoxicity classes (**A**,**B**), sites (**C**,**D**), and species (**E**,**F**) of individual variation in growth, i.e., random effects of non-linear mixed-effects (NLME) modeling of growth) for both von Bertalanffy growth rate coefficient (K) and asymptotic body length ($L\infty$). The plots indicate no detectable relationship between growth and ciguatoxicity or site, and that species effect was well accounted for by the model. See Table 2 for model specification.
